# Prediction of Post-Weaning Fibrinogen Status during Cardiopulmonary Bypass: An Observational Study in 110 Patients

**DOI:** 10.1371/journal.pone.0126692

**Published:** 2015-05-26

**Authors:** Gabor Erdoes, Germaine Gerster, Giuseppe Colucci, Heiko Kaiser, Lorenzo Alberio, Balthasar Eberle

**Affiliations:** 1 Department of Anesthesiology and Pain Therapy, University Hospital Bern, Bern, Switzerland; 2 Clinica Luganese Moncucco, Lugano, Switzerland; 3 Hématologie non-maligne et Hémostase, Service et Laboratoire central d`Hématologie, University Hospital Lausanne, Lausanne, Switzerland; University of Colorado Denver, UNITED STATES

## Abstract

**Background:**

After cardiac surgery with cardiopulmonary bypass (CPB), acquired coagulopathy often leads to post-CPB bleeding. Though multifactorial in origin, this coagulopathy is often aggravated by deficient fibrinogen levels.

**Objective:**

To assess whether laboratory and thrombelastometric testing on CPB can predict plasma fibrinogen immediately after CPB weaning.

**Patients / Methods:**

This prospective study in 110 patients undergoing major cardiovascular surgery at risk of post-CPB bleeding compares fibrinogen level (Clauss method) and function (fibrin-specific thrombelastometry) in order to study the predictability of their course early after termination of CPB. Linear regression analysis and receiver operating characteristics were used to determine correlations and predictive accuracy.

**Results:**

Quantitative estimation of post-CPB Clauss fibrinogen from on-CPB fibrinogen was feasible with small bias (+0.19 g/l), but with poor precision and a percentage of error >30%. A clinically useful alternative approach was developed by using on-CPB A10 to predict a Clauss fibrinogen range of interest instead of a discrete level. An on-CPB A10 ≤10 mm identified patients with a post-CPB Clauss fibrinogen of ≤1.5 g/l with a sensitivity of 0.99 and a positive predictive value of 0.60; it also identified those without a post-CPB Clauss fibrinogen <2.0 g/l with a specificity of 0.83.

**Conclusions:**

When measured on CPB prior to weaning, a FIBTEM A10 ≤10 mm is an early alert for post-CPB fibrinogen levels below or within the substitution range (1.5–2.0 g/l) recommended in case of post-CPB coagulopathic bleeding. This helps to minimize the delay to data-based hemostatic management after weaning from CPB.

## Introduction

Platelet aggregation and hemostatic clot formation are strongly associated with the presence of fibrinogen, a soluble glycoprotein upregulated in a variety of inflammatory conditions.[1–3] Recently, fibrinogen gained renewed clinical interest after a number of studies demonstrated the inverse relationship between fibrinogen levels, perioperative blood loss and exposure to allogeneic blood transfusion.[1–9] In cardiac surgery with cardiopulmonary bypass (CPB), major post-CPB bleeding is not infrequent since CPB-associated conditions (e.g., hemodilution, hypothermia, platelet dysfunction and fibrinolysis) impair fibrinogen levels, function and hence, clot stability. This multifactorial acquired coagulopathy increases bleeding, allogeneic blood product exposure, morbidity and mortality.[5,10–15] Current European guidelines recommend fibrinogen substitution as a first-line therapy in case of significant bleeding accompanied by low levels of plasma fibrinogen.[16,17]

Plasma fibrinogen level is traditionally determined by time-consuming direct measurement (clottable fibrinogen according to the technique by Clauss). More recently, in a point-of-care setting and much faster, thrombelastometry (ROTEM) is used to quantify fibrin-dependent clot firmness (FIBTEM assay). It has been advocated that point-of-care thrombelastometry can be used to replace the Clauss method under perioperative conditions, not only due to its more comprehensive representation of functional fibrinogen potential, but also for its much shorter turnaround time.[18–20] Current guidelines recommend fibrinogen substitution if substantial bleeding is accompanied by a plasma fibrinogen of less than 1.5 to 2.0 g/l.[21–24] Selection of a clinically appropriate substitution threshold and target range, and accurate as well as rapid determination of fibrinogen status around these points are needed for efficacious and safe hemostatic management. Undersubstitution, treatment delays or oversubstitution are all likely to increase complication rate. Undersubstitution will fail to normalize clinical coagulation in due time, resulting in continued blood loss, prolonged hemodynamic instability, difficult surgical hemostasis and exposure to repetitive transfusion rounds. Abnormally high fibrinogen levels, e.g. in the range commonly observed beyond the second postoperative day, are an independent risk factor for thrombosis, stroke and myocardial infarction.[9,25–27]

We hypothesized that analysis of fibrinogen level and function during CPB prior to weaning provides early and reliable information for the immediate post-weaning diagnosis and treatment of hypofibrinemic bleeding. The aim of our study was to compare Clauss fibrinogen levels and simultaneously sampled FIBTEM assays during rewarming prior to CPB weaning as well as after weaning and heparin reversal, and to assess discriminatory power of FIBTEM parameters for post-CPB fibrinogen substitution thresholds recommended by current guidelines [24,28].

## Materials and Methods

### Study Design and Patient Cohort

This is a prospective observational study of sequential coagulation analysis during cardiovascular surgery on CPB. Consecutive patients undergoing a prospectively defined variety of cardiovascular procedures with moderate to high bleeding risk, according to the study of Karkouti et al.,[29] were included, with the aim to provide external validity for a patient population of an academic cardiac center: combined coronary and valve surgery, re-do cardiac surgery, cardiac transplantation, left ventricular assist device implantation, use of hypothermic circulatory arrest, thoracic or thoracoabdominal aortic repair, or with other predictors of increased post-CPB bleeding risk (active dual antiplatelet therapy, liver insufficiency, prolonged CPB runs >120 min). All procedures were performed at the University Hospital Bern, Switzerland, between June 2009 and July 2011.

Since acquisition of ROTEM analysis by our hospital in 2009, institutional policy of the Department of Hematology and Central Hematology Laboratory stipulated availability of sequential, and simultaneously sampled, conventional and thrombelastometric coagulation test results prior to the release of factor concentrates to operating room teams. In order to reduce laboratory expense in the future, these datasets were analyzed for clinically useful correlations and predictive power. All patients undergoing cardiovascular procedures on CPB as mentioned above were screened for the study. Exclusion criteria were unpaired conventional and FIBTEM samples, or exogenous substitution with fibrinogen concentrate during the study period, i.e., pre-, on, and post-CPB until transfer to the intensive care unit (ICU).

Ethical approval for the study (registration number 197/08) was provided by the Cantonal Ethics Committee (Kantonale Ethikkommission Bern, Postfach 56, 3010 Bern. Contact: Dr. sc. nat. Dorothy Pfiffner). All patients gave written informed consent for pertinent laboratory analysis and blood product substitution, and for use of their anonymized data for research purposes. The STROBE checklist for observational studies was used to guide the methods and to structure this manuscript.[30]

### Anesthesia, CPB and Management of Hemostasis

All patients received general anesthesia and cardiovascular surgery according to institutional standards. Anesthetic monitoring included American Society of Anesthesiologists standard monitoring, invasive blood pressure, central venous pressure, urine output and nasopharyngeal temperature monitoring. Depending on the procedure, cardiac surgery was performed using either conventional extracorporeal circulation (CECC; Maquet Holding, Germany; 88% of the patients) primed with 500 ml hydroxyethyl starch (130/0.4), 1000 ml Ringer´s solution, 100 ml of 20% mannitol and 10.000 IU heparin, or a minimized extracorporeal circuit (MECC; Medtronic, Minneapolis, USA; 12% of the patients) primed with 600 ml Ringer´s solution and 5000 IU heparin. No heparin-coated components were used. Routine antifibrinolytic treatment consisted of tranexamic acid (loading dose, 30 mg/kg over 30 min after heparin administration followed by 15 mg/kg/h until skin closure). Myocardial protection was provided with a single dose of 100 ml antegrade crystalloid cardioplegia (Cardioplex, Bichsel AG, Interlaken, Switzerland), and in the case of prolonged bypass time, with additional high-potassium cold blood cardioplegia (Buckberg Solution, Bichsel AG., Interlaken, Switzerland) repeated every 20–30 minutes. A bolus of heparin was given (CECC: 500 IU kg^-1^; MECC: 400 IU kg^-1^) prior to aortic cannulation to achieve a target kaolin-activated clotting time (high-range ACT) of >480 seconds (Activated Coagulation Timer ACT II, Medtronic, Minneapolis, USA) before starting cardiopulmonary bypass. On CECC during full anticoagulation, shed blood was filtered and reinfused via the cardiotomy reservoir into the CPB circuit, or underwent autologous cell salvage, red blood cell separation, processing and reinfusion pre- and post-heparinization. With MECC, shed blood was entirely salvaged using an optoelectrical sensor suction device (SMART, Fumedica AG., Muri, Switzerland), which is activated only when in direct contact with liquid; here, all suctioned blood was first processed using a cell-saving device and then reinfused. After weaning from CPB, heparin was reversed with protamine in a ratio of 1:1 with regard to the initial heparin bolus. A post-reversal ACT of <130 seconds was targeted.

Intraoperative coagulation testing was performed from venous blood sampled from a central venous line or from the extracorporeal circuit and sent by pneumatic tube transport to the central hematology laboratory for complete blood count, international normalized ratio, fibrinogen concentration (Clauss method) and simultaneous thrombelastometry (ROTEM delta, Axon Lab, Switzerland). To account for our laboratory´s turnover time of 45 min for conventional coagulation testing, samples were obtained 30–40 min prior to the projected termination of CPB (on-CPB), and again after full heparin reversal with protamine (post-CPB). Choice and amount of transfused allogeneic blood products were left to the discretion of the attending anesthesiologist and intensivist, respectively, within the limits of our institutional transfusion guidelines. Fresh frozen plasma, which contains fibrinogen, was only given after obtaining blood for the post-CPB measurement time point.

### Measurement of Plasma Fibrinogen

Plasma levels of fibrinogen were measured using the Clauss technique on a commercially available coagulation analyzer (BCS, Dade Behring Inc., Germany) using the Multifibren U-Reagent according to manufacturer`s specifications and pertinent publications.[31] Briefly, excess thrombin added to diluted and recalcified citrated plasma converts fibrinogen into fibrin monomers, which spontaneously polymerize into a clot. The time needed for clot formation is inversely correlated with the plasma fibrinogen, which is calculated using a calibration curve.

Sequential analysis using thrombelastometry included assays for INTEM, EXTEM, FIBTEM and HEPTEM. Briefly, native or—as in our study—recalcified citrated whole blood (300 μl) is immersed into a heated cuvette with an oscillating pin suspended in the blood sample. Rotation of the pin back and forth through an angle of 4°75`in the center of the cuvette initiates platelet activation and fibrin polymerization, which in turn increases torque and progressively restricts pin movement. The viscoelastic change is transmitted for signal processing and graphical display via an optical detector system composed of a light source with a mirror mounted on axis. [32,33] In FIBTEM, tissue factor is used as activator with cytochalasin D added for platelet inhibition, which provides a measure of net fibrinogen polymerization. Although all ROTEM variables were recorded, comparative analysis in this study focuses on clot formation amplitude at 10 min (A10) and maximum clot firmness (MCF). Reference range was 9–24 mm for A10 and 8–25 mm for MCF.[34] In order to ensure quality control and to minimize user-dependent variability, all coagulation and thrombelastometry analyses were performed in our central hematology lab at an analysis temperature of 37°C by certified bioanalytical technicians. Pre-analytical workup, parameters and real-time display of results in the operating room have been described previously.[19,32,35–37]

### Data Collection and Statistical Analyses

Demographic data and intraoperative variables were collected in a prospective observational fashion. Clauss fibrinogen and ROTEM results were stored in and retrieved from the laboratory´s data base system. Analysis time stamps were linked to CPB time stamps, comparing simultaneously acquired Clauss fibrinogen and FIBTEM results sampled prior to (pre-CPB), during (on-CPB) and after CPB (post-CPB).

Linear regression analysis was used to assess the relationship between Clauss fibrinogen and FIBTEM variables, or between different times of measurement using the Pearson product-moment correlation coefficient (Pearson´s r). Methods were compared using mean difference (bias), the standard deviation of the difference and the limits of agreement (bias ± 1.96 x standard deviations), also expressed as the percentage of error (= 100 x 1.96 x standard deviation of the difference between the post-CPB and the on-CPB value / mean of on-CPB and post-CPB value). Receiver operating characteristics (ROC) analysis was used to assess diagnostic or predictive power of FIBTEM and fibrinogen data.

Data are presented as counts (percentage), mean ± standard deviation, median with interquartile ranges [25q;75q], regression coefficient (r) and area under the curve for ROC (AUC) as appropriate. A p-value of <0.05 was considered statistically significant. For statistical analysis we used SigmaPlot for Windows, Version 10.0 and SigmaStat for Windows, Version 3.0 (Systat Software, Inc., Germany).

## Results

### Procedural and Outcome Data

Of 2232 CPB procedures in the observation period, 110 patients had paired conventional and FIBTEM samples, and did not receive exogenous substitution with fibrinogen concentrate during the study period, i.e., pre-, on, and post-CPB until transfer to the intensive care unit (ICU). Demographics and preoperative anticoagulant medication are given in Table 1.

**Table 1:** Patients`demographics and preoperative antithrombotic medication.

Age, years	62 ± 14
Female sex	31 (28)
BMI, kg/m^2^	41 ± 4
EuroScore, additive	7 ± 3
Ejection fraction, %	55 ± 14
Pre-operative antithrombotics	
Acetylsalicylic acid	50 (45)
Warfarin	24 (22)
Clopidogrel	14 (13)
Heparin	12 (11)
Nadroparin	8 (7)
Tirofiban	1 (1)
Prasugrel	1 (1)

BMI indicates body mass index. Data are numbers (percent), mean ± standard deviation or median with interquartile ranges [25q;75q] where appropriate.

Table 2 lists procedural data pertinent to perioperative fibrinogen kinetics, and basic outcomes. In this cohort with moderate to high bleeding risk and prolonged duration of CPB, Clauss fibrinogen levels below the institutional lower reference limit (1.75 g/l) were observed in 29% of included patients during CPB, and in 19% after CPB weaning. Allogeneic product transfusion in exposed patients after weaning from CPB until transfer to the ICU were 4 [2;6] units of red blood cells and 4 [2;6] units of fresh frozen plasma (Table 2). Overall perioperative exposure to any allogeneic blood product transfusion was 86% (95 / 110).

**Table 2:** Procedural data.

Procedure	
Isolated CABG	13 (12)
Valve surgery and CABG	39 (35)
Thoracic aorta repair	27 (25)
Thoracoabdominal aorta repair	21 (19)
Heart transplantation and LVAD	10 (9)
Re-do surgery	34 (31)
Emergency surgery	10 (9)
Duration of surgery, min	281 [62;890]
CPB time, min	137 [21;507]
Minimal temperature, Celsius	32 [20;37]
Fluids, coagulants, anticoagulants and transfusion	
RBC concentrate, units	4 [2;6]
Patients exposed to RBC	78 (71)
AWRC, ml	560 [453;696]
Patients exposed to AWRC	77 (70)
FFP, U	4 [2;6]
Patients exposed to FFP	52 (47)
PC, U	2 [1;2]
Patients exposed to PC	60 (55)
PCC, U	11 [1125;1950]
Patients exposed to PCC	10 (9)
Fibrinogen concentrate, g	0 [0;0]
Patients exposed to fibrinogen concentrate	0 (0)
Colloids, ml	0 [0;2100]
Postprocedural chest tube drainage, ml	
After 12h	420 [60;4450]
After 24h	700 [90;5335]
After 48h	800 [90;8010]
Total in ICU	820 [90;26890]
Outcome and complications	
ICU stay, d	1 [1;3]
Extubation, d	1 [0;1]
Hospital discharge, d	12 [8;18]
In-Hospital	
Mortality	11 (10)
Infection	24 (22)
Thromboembolism	11 (10)
Bleeding requiring reexploration	6 (5)

CABG indicates coronary artery bypass grafting; LVAD, left ventricular assist device; CPB, cardiopulmonary bypass; RBC, red blood cell; AWRC, autologous washed red cells; FFP, fresh frozen plasma; PC, platelet concentrate; PCC, prothrombin complex concentrate; ICU, intensive care unit; d, days; h, hours; U, units (1 unit corresponds to 250–275 ml). Data are numbers (percent), mean ± standard deviation or median with interquartile ranges [25q;75q] where appropriate.

During ICU stay, cumulative postoperative chest tube drainage volume was 820 [90;26890] ml. Six patients (5%) required surgical re-exploration due to excessive postoperative bleeding in the ICU. Length of ICU stay was 1 [1;3] days, with readiness for hospital discharge after 12 [8;18] days. Infective and thromboembolic events occurred in 24 (22) and 11 (10) patients, respectively. In-hospital mortality was 10% (11 / 110).

### Changes of Clauss Fibrinogen and FIBTEM in Relation to CPB

**Fig 1:**
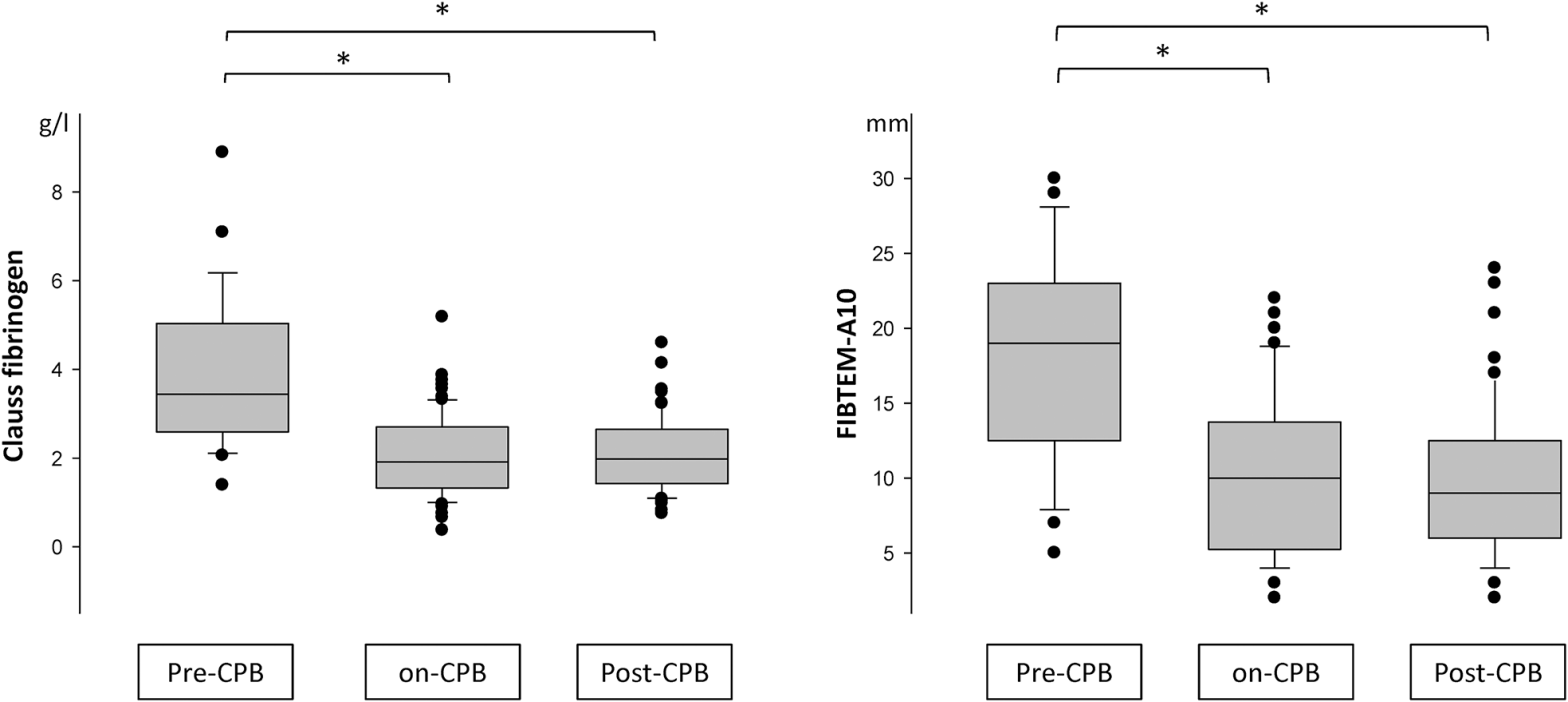
Clauss fibrinogen and A10 in relation to CPB. Boxplot, median, 25^th^/75^th^ percentile; whiskers, 10^th^/90^th^ percentile; extremes. A10, A10. *) denotes significant (p < 0.05) change between times of measurement.

Clauss fibrinogen level and A10 amplitude were highest pre-CPB (fibrinogen, 3.9 ± 1.8 g/l; A10, 18.2 ± 7.1 mm; MCF, 20.1 ± 8.1 mm). On CPB prior to weaning, a significant decrease was apparent (fibrinogen, 2.1 ± 0.9 g/l; A10, 10.2 ± 5.4 mm; MCF, 11.5 ± 6.5 mm), without significant difference to levels early after CPB and heparin reversal (fibrinogen, 2.1 ± 0.8 g/l; A10, 9.7 ± 4.9 mm; MCF, 10.9 ± 5.7 mm) (Fig 1).

### Correlation and Discriminatory Power of Fibrinogen and FIBTEM

**Fig 2:**
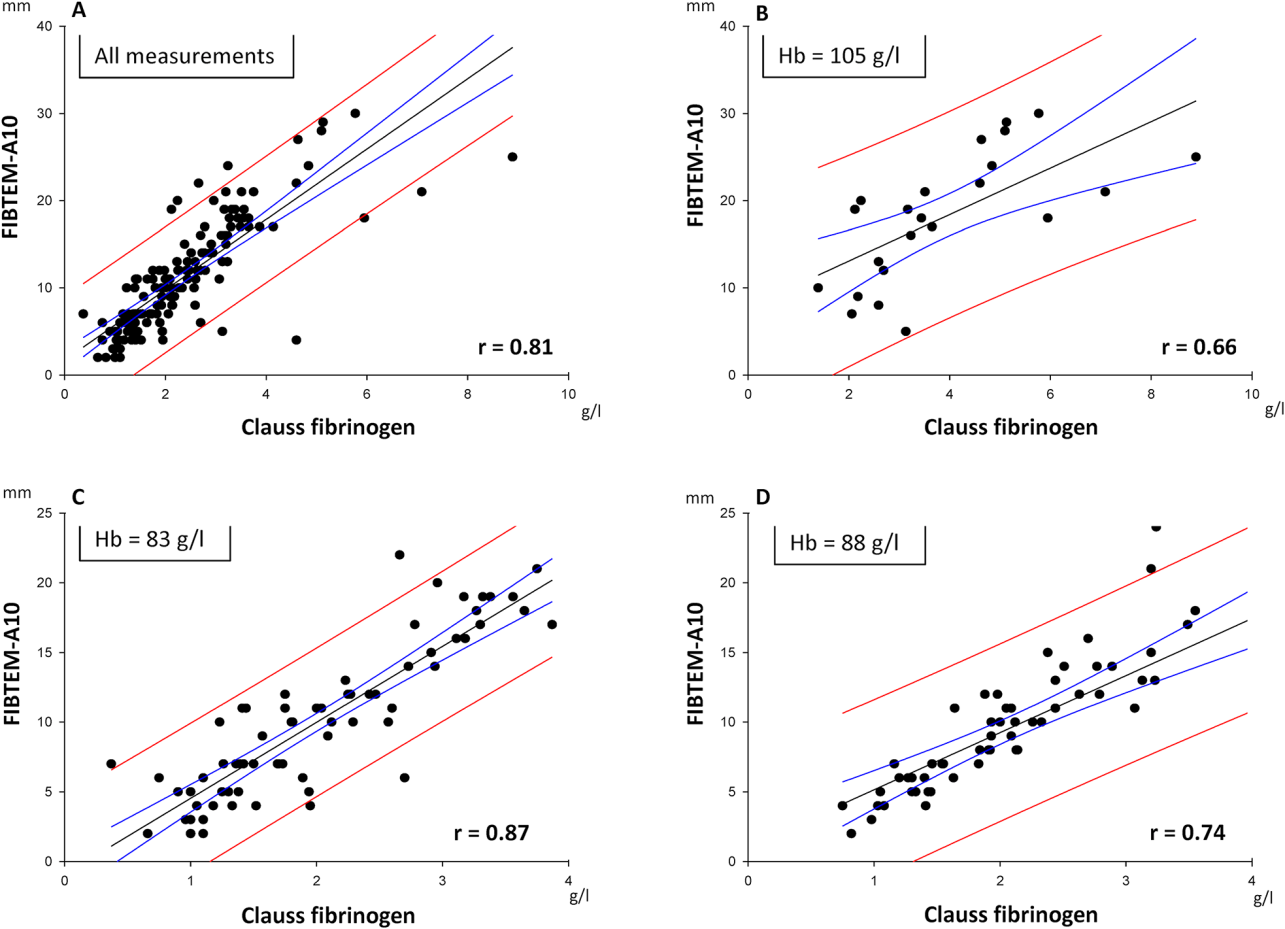
Relationship between Clauss fibrinogen and A10 according to operative period and mean hemoglobin. Regression analysis. Equation: Polynomial, linear. Blue lines = 95% Confidence Band; Red lines = 95% Prediction Band. A10, A10.

Clauss fibrinogen and A10 were significantly correlated (all data points, r = 0.81; p<0.05). Their correlation was closer on-CPB at a mean Hb of 83 g/l (r = 0.87) and post-CPB (mean Hb 88 g/l; r = 0.74) than pre-CPB (mean Hb 105 g/l; r = 0.66) (Fig 2).

**Fig 3:**
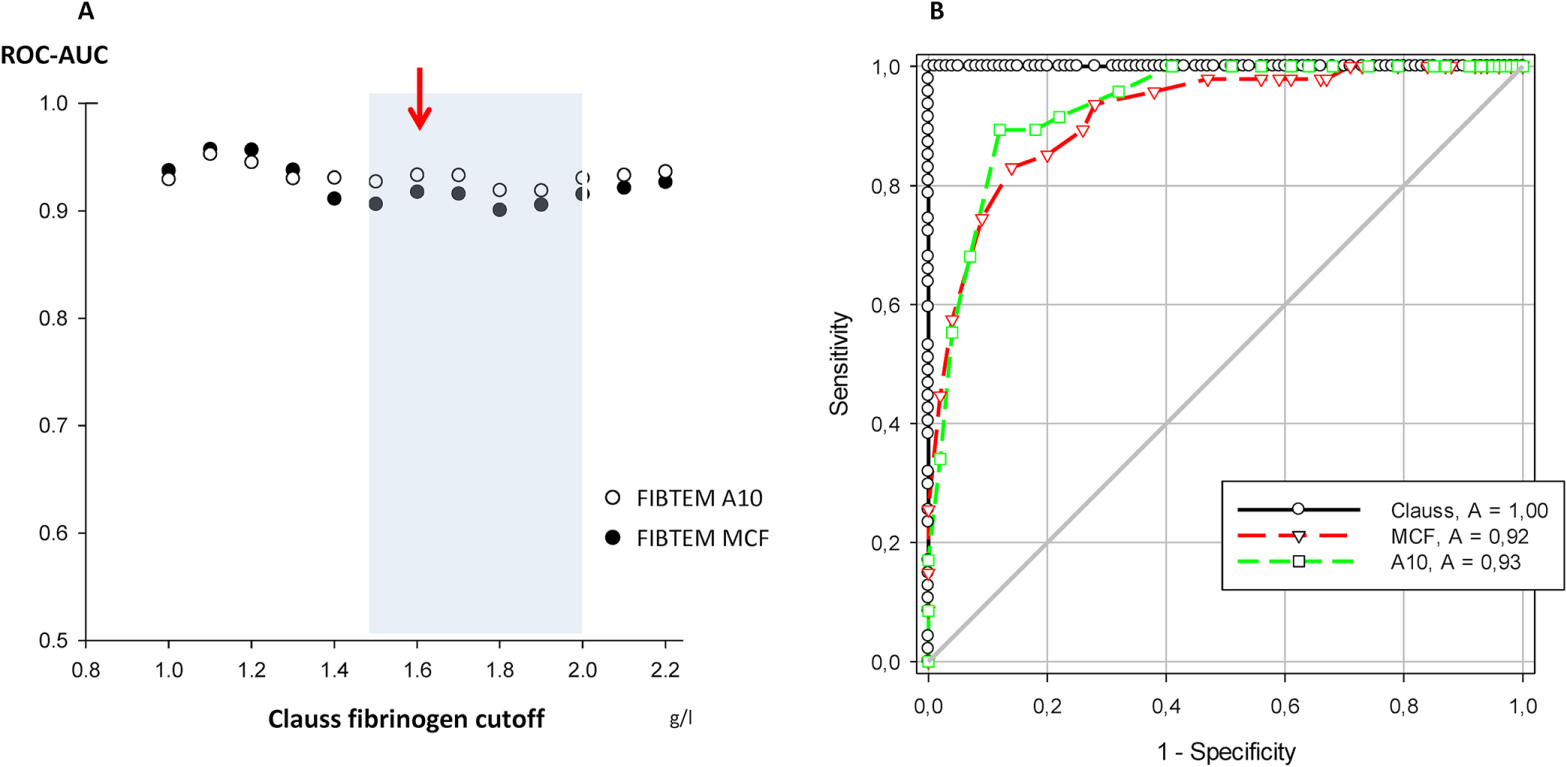
Discriminative power of FIBTEM variables for Clauss fibrinogen (A) and ROC curves at Clauss fibrinogen cutoff of 1.6 g/l (B). Red arrow = highest ROC-AUC in the recommended range for fibrinogen substitution by guidelines (blue transparent square). ROC, receiver operating characteristics.

The discriminatory power of A10 and MCF for various assumed fibrinogen substitution thresholds was determined for fibrinogen cutoffs between 1.0 and 2.2 g/l. ROC analysis showed that FIBTEM discrimination of fibrinogen cutoffs in this range was generally good with some variability (A10, ROC-AUC 0.91 to 0.95; MCF, 0.90 to 0.95 (Fig 3)). For cutoffs between 1.5 g/l and 2.0 g/l, which reflect current recommendations for perioperative fibrinogen substitution [9,16,22,38], the discriminatory power of the more rapid A10 result was not inferior to MCF. Sensitivity and specificity of A10 were balanced best at 7.5 mm, offering an even better maximum sensitivity (0.89) and specificity (0.88) for a fibrinogen cutoff at 1.6 g/l than MCF (Fig 3).

### Within-Test Correlation between On-CPB and Post-CPB Fibrinogen and FIBTEM

**Fig 4:**
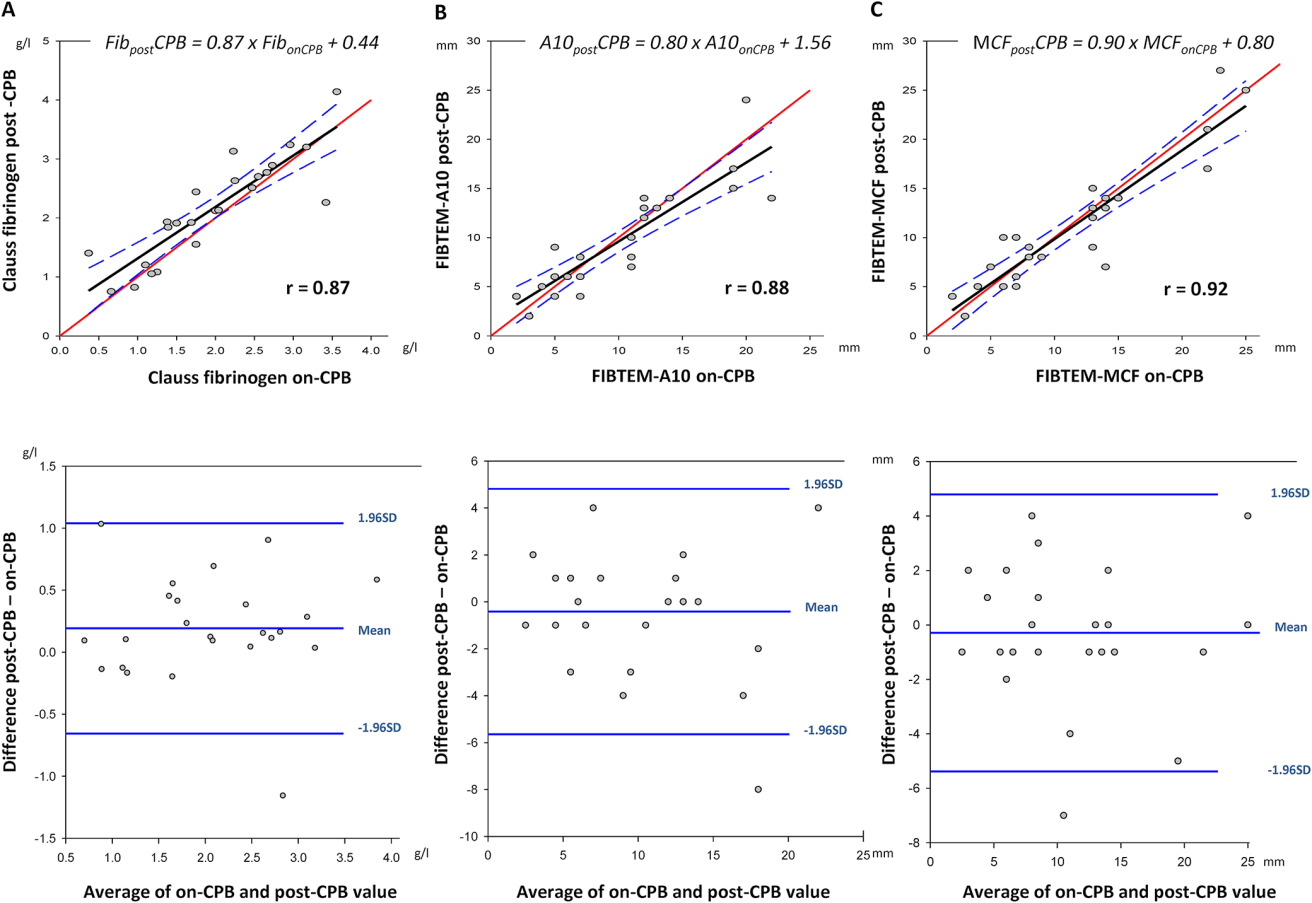
Relationship and correlation estimates between on-CPB and post-CPB levels of Clauss fibrinogen (A), A10 (B) and MCF (C) with corresponding Bland-Altman plot. CPB, cardiopulmonary bypass; FIBTEM A10, A10; MCF, maximum clot firmness.

The difference between on-CPB and post-CPB results for Clauss fibrinogen, A10, or MCF was not significant (Fig 1). Although comparison of measurements also demonstrated close within-test correlation (r > 0.87) and small bias, precision was low and percentage of error was >30% for Clauss fibrinogen, A10 and MCF (Table 3, Fig 4).

**Table 3:** Percentage of error calculation.

	Clauss fibrinogen (g/l)	FIBTEM A10 (mm)	FIBTEM MCF (mm)
Bias	0.19	- 0.42	- 0.29
Standard deviation	0.43	2.67	2.59
Limits of agreement	-0.66, 1.04	-5.64, 4.81	-5.38, 4.79
Bias 95% of confidence interval	0.01–0.37	-1.55–0.71	-1.39–0.81
Percentage of error, %	41.3	53.2	45.3

Percentage of error = 100 x 1.96 x standard deviation of the difference between the post-CPB and the on-CPB value / mean of on-CPB and post-CPB value.

### On-CPB FIBTEM to Predict post-CPB Fibrinogen Substitution Range

**Fig 5:**
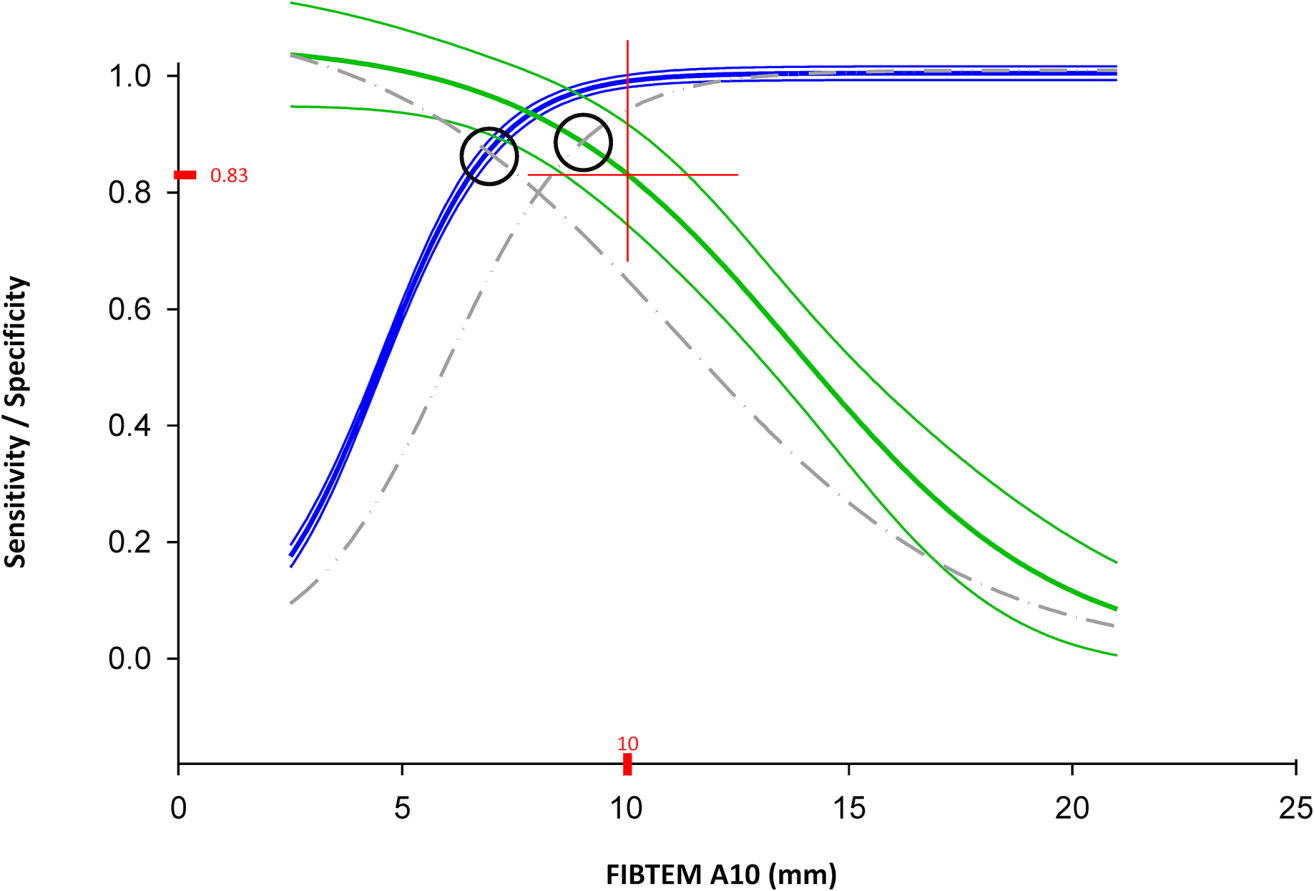
Sensitivity and specificity of A10 for Clauss fibrinogen limits of ≤ 1.5 and ≤ 2.0 g/l, respectively, with optimal operating point at 10 mm (A10). Two-graph fitted ROC curves with 95% confidence interval showing sensitivity and specificity for each limit. Sensitivity (blue curve) and specificity (gray dash-dot curve) for post-CPB Clauss fibrinogen ≤1.5 g/l and sensitivity (gray dash-dot-dot curve) and specificity (green curve) for post-CPB Clauss fibrinogen >2.0 g/l. Black circles: best sensitivity and specificity for Clauss fibrinogen ≤1.5 g/l (left circle, corresponds to A10 of 6.9 mm) and for Clauss fibrinogen >2.0 g/l (right circle, corresponds to A10 of 9.0). Red cross corresponds to optimal operating point with an on-CPB A10 sensitivity for post-CPB Clauss fibrinogen ≤1.5 g/l of 0.99 and a specificity for post-CPB Clauss fibrinogen >2.0 g/l of 0.83.

ROC analysis showed that a Clauss fibrinogen level ≤1.5 g/l is to be expected post-CPB when A10 is below 6.9 mm on-CPB (both sensitivity and specificity = 0.87). Likewise, a post-CPB fibrinogen >2.0 g/l is likely when on-CPB A10 amplitude is above 9.0 mm (both sensitivity and specificity = 0.88) (black circles, Fig 5).

This diagnostic accuracy, however, is still less than satisfactory. For clinical purposes, it is more helpful to rapidly pre-identify all patients with low fibrinogen post-CPB (i.e., aiming for high sensitivity of A10 on-CPB for post-CPB fibrinogen ≤1.5 g/l). On the other hand, exogenous fibrinogen administration appears unreasonable at post-CPB fibrinogen levels ≥2.0 g/l. Thus, on-CPB prediction using A10 should aim for high specificity for post-CPB fibrinogen <2.0 g/l. These two clinically relevant conditions were fulfilled best at an on-CPB A10 of 10 mm, with sensitivity (lower border of substitution range) at 0.99, and specificity (upper border) at 0.83, respectively (Fig 5). This on-CPB A10 amplitude had a positive predictive value (PPV) of 0.60 for post-CPB fibrinogen ≤ 1.5 g/l and a negative predictive value (NPV) of 0.92 for post-CPB fibrinogen <2.0 g/l.

### Intraoperative Allogeneic Blood Product Use and Postoperative Drainage Loss

Post-CPB fibrinogen assessment was followed by significant use of fresh frozen plasma, platelets and an overall high exposure to allogeneic transfusion in our study population (Table 2). Subsets of patients with on-CPB A10 ≤10 mm and A10 >10 mm in the post-CPB period did not show statistically significant difference for transfusion of any blood product (p values for red blood cells = 0.35; fresh frozen plasma = 0.07; platelet concentrate = 0.19) or for chest tube loss (after 24 hours: p = 0.34).

## Discussion

This observational study tracked conventional and thrombelastometric fibrinogen status on weaning from CPB in a representative cohort undergoing major cardiovascular surgery. Its main findings were as follows:

In accordance with the current rationale for thrombelastometry in suspected hypofibrinogenemic post-CPB bleeding, FIBTEM A10 and MCF correlate with plasma fibrinogen (Clauss). This is more marked with hemodilution, as has been described before [39]. FIBTEM A10 is more rapidly available, and discriminates equally well or better than MCF in the range of clinical interest between 1.5 g/l and 2.0 g/l plasma fibrinogen. Also, pre-weaning and post-CPB results of both techniques are closely correlated, which can be used for planning of post-CPB hemostatic management. Despite such significant correlations, however, there is considerable imprecision both in FIBTEM-based fibrinogen estimates and in prediction of post-CPB values from pre-weaning data. By choosing a categorical approach instead, we found that FIBTEM A10, when sampled on CPB prior to weaning, allows accurate and clinically useful identification of patients who will be in- or outside a target zone of 1.5–2.0 g/l plasma fibrinogen after weaning and heparin reversal.

Our findings of correlations between conventional and point-of-care fibrinogen test methods are confirmative of current literature for a large and clinically representative variety of major cardiovascular surgery.[32,40,41] However, even if Clauss fibrinogen and FIBTEM correlate with each other, and also show close within-test correlation and small bias, the limits of agreement between on-CPB and post-CPB samples are wide. An exact prediction of the latter, e.g., by using linear regression equations of Fig 4, is thus not possible. Therefore we chose a categorical approach [42], classifying predicted post-CPB fibrinogen into three clinically relevant zones of a) hypofibrinogenemia ≤1.5 g/l, b) a target range of 1.6–2.0 g/l, and c) fibrinogen >2.0 g/l. We identified an optimal operating point at an A10 of 10 mm, measured on-CPB prior to weaning. This decision threshold provides a sensitivity of 0.99 for ruling in a post-CPB fibrinogen ≤1.5 g/l (Clauss method). Thus, in case of non-surgical bleeding after heparin reversal, such information is already at hand for triggering fibrinogen substitution towards the target range of 1.5–2.0 g/l.[9,16,22,38] The same threshold allows on CPB to rule out a post-CPB fibrinogen >2.0 g/l with a specificity of 0.83, in order to minimize oversubstitution in conjunction with clinical assessment of bleeding. A lower diagnostic resolution of on-CPB A10 for fibrinogen cutoff at 1.5 g/l (positive predictive value 0.60) appears clinically acceptable in view of the lack of safety signals for fibrinogen replacement (with FFP or factor concentrate) at levels below 2.0 g/l.[43] With this approach, and regardless of whether conventional or point-of-care testing is the preferred institutional routine, on-CPB sampling during rewarming enables the clinician to plan post-CPB fibrinogen management in a timely and simple fashion. Thus early diagnosis and immediate, data-driven treatment of hypofibrinogenemic bleeding after termination of CPB is feasible.

Our study, although observational and from a single institution, has external validity since it analyses a mixed cohort of patients undergoing major cardiovascular surgery at risk of post-CPB bleeding, transfusion and mortality, and without exogenously administered fibrinogen concentrate.

At the time of the study, fibrinogen concentrate was released by our Department of Hematology for perioperative replacement exclusively after documentation of post-CPB fibrinogen levels (Clauss) of 1.0 g/l or less, in accordance with 2006 ASA guidelines.[38] Applying the decision rule derived from this study, 40% of our patients would have received exogenous fibrinogen substitution based on an A10 ≤10 mm on-CPB. Instead, in our cohort, post-CPB fibrinogen assessment was followed by significant use of fresh frozen plasma, platelets and an overall high exposure to allogeneic transfusion in our study population. This is a likely explanation why postoperative chest tube drainage did not differ between subsets of patients with on-CPB A10 ≤10 mm and A10 >10 mm (p = 0.34 for chest tube drainage loss after 24 hours). Whether early FIBTEM-guided administration of fibrinogen concentrate in above 40% of patients at risk would have resulted in a reduction of blood loss and allogeneic transfusion exposure, is to be clarified by further studies.

Low levels of plasma fibrinogen appear associated with excessive blood loss and poor patient outcome after cardiac surgery.[4,7–9,14,21,44,45] It has been also demonstrated that after termination of CPB, plasma fibrinogen levels reach an individual nadir and tend to recover until 24 hours postoperatively.[8,13,46,47] Taken together, hemostasis in the early post-CPB period may be compromised substantially by abnormally low plasma fibrinogen, with consequences like excessive or protracted blood loss, impaired exposure during surgical hemostasis, prolonged systemic hypoperfusion, hemodilution, ionized hypocalcaemia, hypothermia and lactic acidosis. Compensatory use of vasopressors, inotropes and allogeneic blood products are associated with, or may be even causative for increased perioperative morbidity and mortality. Despite the well-documented importance of sufficient plasma fibrinogen levels in the immediate post-CPB period, there are still conflicting recommendations about suitable trigger levels for fibrinogen substitution.[21–23]. A plasma level of 0.8–1 g/l is nowadays regarded as a too low a trigger for exogenous fibrinogen replacement.[9,10] On the other end of the spectrum, a fibrinogen target of >3.0 g/l, as suggested by some authors,[16] does not produce supranormal ROTEM parameters *in vitro*.[22] It may even cause harm *in vivo* by promoting thromboembolic cardiovascular events and increasing medical cost.[16,27,48] At present, a target range of 1.5–2.0 g/l for fibrinogen substitution during coagulopathic bleeding is recommended.[8,17, 23,38, 49–51]

Cardiac surgery is characterized by acute, but usually foreseeable and often iatrogenic perturbations of the coagulation system by anticoagulants, hemodilution, surface activation and hypothermia. Thus, early prediction of post-CPB fibrinogen status is an appealing idea. So far, this approach has been neglected in favor of prophylactic indiscriminate use of antifibrinolytics, and of labor-intensive point-of-care testing.[52] Only recently, Engberink et al. prospectively investigated the diagnostic value of early ROTEM amplitudes for thrombocytopenia and hypofibrinogenemia in cardiac surgery.[53] They found that FIBTEM-A5, providing results after 5 min of initialization, closely correlates to A10 (r = 1.00), MCF (r = 0.99) and Clauss fibrinogen (r = 0.87). Correlation between simultaneously sampled FIBTEM and fibrinogen data in this study is very similar to our results. By replacing A10 with A5, the authors were able to estimate concomitant fibrinogen levels 5 minutes earlier with similar diagnostic accuracy. Görlinger et al. confirmed the good correlation of early FIBTEM variables (A5, A10, A15) with MCF in non-cardiac procedures.[18] When comparing Clauss fibrinogen test performance between different laboratories, Solomon et al. found no change in plasma fibrinogen when measured before and after CPB weaning (varying from -0.17 to 0.13, depending on center) and hypothesized that Clauss measurements on CPB could allow early estimation of fibrinogen deficit after weaning.[54]

Our study confirms this hypothesis in a representative cardiac surgical population with a variable degree of acquired hypofibrinogenemia. However, even if performed by certified lab technicians, pre-weaning estimation of post-protamine fibrinogen parameters lacks precision, according to Bland-Altman analysis of our data. We therefore propose to omit the idea of point-to-point prediction in favor of a categorical approach. Our results show that Clauss fibrinogen, FIBTEM parameters, or the combination of both, when sampled on CPB, allow accurate and clinically useful identification of patients who will be below or within a target zone of 1.5–2.0 g/l plasma fibrinogen after weaning and heparin reversal. In our study, an A10 ≤10 mm on CPB classified a patient either as hypofibrinogenemic (99% sensitivity for ≤1.5 g/l) or within the substitution range (83% specificity for <2.0 g/l) following heparin reversal. This allows timely preparation of fibrinogen replacement for patients bleeding non-surgically after CPB, but avoids overtreatment with fibrinogen concentrate, particularly so when bleeding can be stopped surgically.

Our observational study has several limitations. Data come from a single center and from a heterogeneous group of patients. This heterogeneity of the cohort with only few patients in some of the subgroups (e.g. isolated CABG, thoracoabdominal aorta repair) may be seen as disadvantage. However, this composition of surgical therapies reflects a realistic clinical spectrum for university-based cardiac surgical services. Thus, we believe that the heterogeneity of our study cohort adds to external validity of our decision strategy for fibrinogen substitution. Still, prediction accuracy and decision thresholds may vary depending on center, laboratory, Clauss assay, ROTEM operator, but also for different patient populations, surgical and perfusion techniques. [54] For instance, the Multifibren U reagent used in our Clauss analysis is known to be potentially sensitive to the high heparin levels (>2 IU/ml) routinely used on CPB.[54,55] This, or alternatively hemoconcentration at the end of the CPB run, might explain the numerical, though insignificant, increase in post-CPB fibrinogen observed by us and others.[54,55] Also, the exact time point of paired sampling prior to CPB weaning, and hence, heparin concentration in the circuit, could not be strictly standardized in relation to the termination of the CPB run. Such potential confounders are an argument to prefer, on-CPB prior to weaning, a FIBTEM-based predictor instead. Finally, perioperative use of hydroxyethyl starch varied somewhat depending on CPB type used (ECC, MECC). This may have affected both fibrinogen and FIBTEM test accuracy [56–58], i.e., by causing overestimation of fibrinogen in Clauss testing, and underestimation of true activity by FIBTEM amplitudes, thus contributing to imprecision of FIBTEM-derived post-CPB fibrinogen estimates.[56–58] Overall however, the dose of colloid in our series was much lower than reported in comparable studies (Table 2).[59,60] Since hydroxyethyl starch is about to disappear as a confounder of intraoperative coagulation testing, predictive accuracy may improve in future analyses.

In conclusion, our study describes a clinically useful decision approach for early prediction of post-CPB fibrinogen status from thrombelastometry obtained on CPB prior to weaning. It allows clinicians a timely and accurate identification of patients who will, after weaning and heparin reversal, present with plasma fibrinogen below or within the range of 1.5–2.0 g/l recommended as substitution target by current guidelines.

## References

[1] Kreuz W, Meili E, Peter-Salonen K, Dobrkovská A, Devay J, Haertel S, et al Pharmacokinetic properties of a pasteurised fibrinogen concentrate. Transfus Apher Sci. 2005;32: 239–46. 10.1016/j.transci.2004.04.003 15919241

[2] Mosesson MW, Siebenlist KR, Meh DA. The structure and biological features of fibrinogen and fibrin. Ann N Y Acad Sci. 2001;936: 11–30. 11460466 10.1111/j.1749-6632.2001.tb03491.x

[3] Davalos D, Akassoglou K. Fibrinogen as a key regulator of inflammation in disease. Semin Immunopathol. 2012;34: 43–62. 10.1007/s00281-011-0290-8 22037947

[4] Solomon C, Pichlmaier U, Schoechl H, Hagl C, Raymondos K, Scheinichen D, et al Recovery of fibrinogen after administration of fibrinogen concentrate to patients with severe bleeding after cardiopulmonary bypass surgery. Br J Anaesth. 2010;104: 555–62. 10.1093/bja/aeq058 20348140 PMC2855672

[5] Karlsson M, Ternström L, Hyllner M, Baghaei F, Nilsson S, Jeppsson A. Plasma fibrinogen level, bleeding, and transfusion after on-pump coronary artery bypass grafting surgery: a prospective observational study. Transfusion. 2008;48: 2152–58. 10.1111/j.1537-2995.2008.01827.x 18657083

[6] Tanaka KA, Egan K, Szlam F, Ogawa S, Roback JD, Sreeram G, et al Transfusion and hematologic variables after fibrinogen or platelet transfusion in valve replacement surgery: preliminary data of purified lyophilized human fibrinogen concentrate versus conventional transfusion. Transfusion. 2014;54: 109–18. 10.1111/trf.12248 23718572

[7] Waldén K, Jeppsson A, Nasic S, Backlund E, Karlsson M. Low Preoperative Fibrinogen Plasma Concentration Is Associated With Excessive Bleeding After Cardiac Operations. Ann Thorac Surg. 10.1016/j.athoracsur.2013.11.064 24507940

[8] Faraoni D, Willems A, Savan V, Demanet H, De Ville A, Van der Linden P. Plasma fibrinogen concentration is correlated with postoperative blood loss in children undergoing cardiac surgery: A retrospective review. Eur J Anaesthesiol. 10.1097/EJA.0000000000000043 24503704

[9] Karkouti K, Callum J, Crowther MA, McCluskey SA, Pendergrast J, Tait G, et al The relationship between fibrinogen levels after cardiopulmonary bypass and large volume red cell transfusion in cardiac surgery: an observational study. Anesth Analg. 2013; 117: 14–22. 10.1213/ANE.0b013e318292efa4 23687229

[10] Davidson S. State of the Art—How I manage coagulopathy in cardiac surgery patients. Br J Haematol. 10.1111/bjh.12746 24450971

[11] Despotis GJ, Avidan MS, Hogue CW. Mechanisms and attenuation of hemostatic activation during extracorporeal circulation. Ann Thorac Surg. 2001;72: S1821–31. 11722116 10.1016/s0003-4975(01)03211-8

[12] Paparella D, Brister SJ, Buchanan MR. Coagulation disorders of cardiopulmonary bypass: a review. Intensive Care Med. 2004;30: 1873–81. 10.1007/s00134-004-2388-0 15278267

[13] Blome M, Isgro F, Kiessling AH, Skuras J, Haubelt H, Hellstern P, et al Relationship between factor XIII activity, fibrinogen, haemostasis screening tests and postoperative bleeding in cardiopulmonary bypass surgery. Thromb Haemost. 2005;93: 1101–07. 10.1267/THRO05061101 15968395

[14] Ucar HI, Oc M, Tok M, Dogan OF, Oc B, Aydin A, et al Preoperative fibrinogen levels as a predictor of postoperative bleeding after open heart surgery. Heart Surg Forum. 2007;10: E392–96. 10.1532/HSF98.20071065 17855205

[15] Ogawa S, Ohnishi T, Hosokawa K, Szlam F, Chen EP, Tanaka KA. Haemodilution-induced changes in coagulation and effects of haemostatic components under flow conditions. Br J Anaesth. 2013;111: 1013–23. 10.1093/bja/aet229 23794670

[16] Kozek-Langenecker SA, Afshari A, Albaladejo P, Santullano CAA, De Robertis E, Filipescu DC, et al Management of severe perioperative bleeding: guidelines from the European Society of Anaesthesiology. Eur J Anaesthesiol. 2013;30: 270–82. 10.1097/EJA.0b013e32835f4d5b 23656742

[17] Spahn DR, Bouillon B, Cerny V, Coats TJ, Duranteau J, Fernández-Mondéjar E, et al Management of bleeding and coagulopathy following major trauma: an updated European guideline. Crit Care. 2013;17: R76 10.1186/cc12685 23601765 PMC4056078

[18] Görlinger K, Dirkmann D, Solomon C, Hanke AA. Fast interpretation of thromboelastometry in non-cardiac surgery: reliability in patients with hypo-, normo-, and hypercoagulability. Br J Anaesth. 2013;110: 222–30. 10.1093/bja/aes374 23112213

[19] Ogawa S, Szlam F, Chen EP, Nishimura T, Kim H, Roback JD, et al A comparative evaluation of rotation thromboelastometry and standard coagulation tests in hemodilution-induced coagulation changes after cardiac surgery. Transfusion. 2012;52: 14–22. 10.1111/j.1537-2995.2011.03241.x 21756263

[20] Solomon C, Cadamuro J, Ziegler B, Schöchl H, Varvenne M, Sørensen B, et al A comparison of fibrinogen measurement methods with fibrin clot elasticity assessed by thromboelastometry, before and after administration of fibrinogen concentrate in cardiac surgery patients. Transfusion. 2011;51: 1695–1706. 10.1111/j.1537-2995.2011.03066.x 21352237

[21] Rahe-Meyer N. Fibrinogen concentrate in the treatment of severe bleeding after aortic aneurysm graft surgery. Thromb Res. 2011;128 Suppl 1: S17–S19. 10.1016/S0049-3848(12)70005-1 22221846

[22] Bolliger D, Szlam F, Molinaro RJ, Rahe-Meyer N, Levy JH, Tanaka KA. Finding the optimal concentration range for fibrinogen replacement after severe haemodilution: an in vitro model. Br J Anaesth. 2009;102: 793–99. 10.1093/bja/aep098 19420005

[23] Rossaint R, Bouillon B, Cerny V, Coats TJ, Duranteau J, Fernández-Mondéjar E, et al Management of bleeding following major trauma: an updated European guideline. Crit Care. 2010;14: R52 10.1186/cc8943 20370902 PMC2887168

[24] Spahn DR, Bouillon B, Cerny V, Coats TJ, Duranteau J, Fernández-Mondéjar E, et al Management of bleeding and coagulopathy following major trauma: an updated European guideline. Crit Care. 2013;17: R76 10.1186/cc12685 23601765 PMC4056078

[25] Swarowska M, Janowska A, Polczak A, Klimkowicz-Mrowiec A, Pera J, Slowik A, et al. The Sustained Increase of Plasma Fibrinogen During Ischemic Stroke Predicts Worse Outcome Independently of Baseline Fibrinogen Level. Inflammation. 10.1007/s10753-014-9838-9 PMC4077303 24531853

[26] Lang Q, Zhou M, Feng H, Guo J, Chen N, He L. Research on the relationship between fibrinogen level and subtypes of the TOAST criteria in the acute ischemic stroke. BMC Neurol. 2013;13: 207 10.1186/1471-2377-13-207 24354692 PMC3878231

[27] Sabater-Lleal M, Huang J, Chasman D, Naitza S, Dehghan A, Johnson AD, et al Multiethnic meta-analysis of genome-wide association studies in >100 000 subjects identifies 23 fibrinogen-associated Loci but no strong evidence of a causal association between circulating fibrinogen and cardiovascular disease. Circulation. 2013;128: 1310–24. 10.1161/CIRCULATIONAHA.113.002251 23969696 PMC3842025

[28] Kozek-Langenecker SA, Afshari A, Albaladejo P, Santullano CAA, De Robertis E, Filipescu DC, et al Management of severe perioperative bleeding: guidelines from the European Society of Anaesthesiology. Eur J Anaesthesiol. 2013;30: 270–82. 10.1097/EJA.0b013e32835f4d5b 23656742

[29] Karkouti K, McCluskey SA, Syed S, Pazaratz C, Poonawala H, Crowther MA. The influence of perioperative coagulation status on postoperative blood loss in complex cardiac surgery: a prospective observational study. Anesth Analg. 2010;110: 1533–40. 10.1213/ANE.0b013e3181db7991 20435945

[30] Elm von E, Altman DG, Egger M, Pocock SJ, Gøtzsche PC, Vandenbroucke JP, et al The Strengthening the Reporting of Observational Studies in Epidemiology (STROBE): guidelines for reporting observational studies. PLoS Med. 2007; 4: e296 17941714 10.1371/journal.pmed.0040296 PMC2020495

[31] CLAUSS A. [Rapid physiological coagulation method in determination of fibrinogen]. Acta Haematol. 1957;17: 237–46. 13434757 10.1159/000205234

[32] Ganter MT, Hofer CK. Coagulation monitoring: current techniques and clinical use of viscoelastic point-of-care coagulation devices. Anesth Analg. 2008;106: 1366–75. 10.1213/ane.0b013e318168b367 18420846

[33] Bolliger D, Seeberger MD, Tanaka KA. Principles and practice of thromboelastography in clinical coagulation management and transfusion practice. Transfus Med Rev. 2012; 26: 1–13. 10.1016/j.tmrv.2011.07.005 21872428

[34] Lang T, Bauters A, Braun SL, Pötzsch B, Pape von KW, Kolde HJ, et al Multi-centre investigation on reference ranges for ROTEM thromboelastometry. Blood Coagul Fibrinolysis. 2005;16: 301–10. 15870552 10.1097/01.mbc.0000169225.31173.19

[35] Tanaka KA, Bolliger D, Vadlamudi R, Nimmo A. Rotational thromboelastometry (ROTEM)-based coagulation management in cardiac surgery and major trauma. J Cardiothorac Vasc Anesth. 2012;26: 1083–93. 10.1053/j.jvca.2012.06.015 22863406

[36] Colucci G, Giabbani E, Barizzi G, Urwyler N, Alberio L. Laboratory-based ROTEM analysis: implementing pneumatic tube transport and real-time graphic transmission. Int J Lab Hematol. 2011;33: 441–46. 10.1111/j.1751-553X.2011.01303.x 21382181

[37] Amann G, Zehntner C, Marti F, Colucci G. Effect of acceleration forces during transport through a pneumatic tube system on ROTEM analysis. Clin Chem Lab Med. 2012;50: 1335–42. 10.1515/cclm-2011-0800 22868797

[38] Bolliger D, Görlinger K, Tanaka KA. Pathophysiology and treatment of coagulopathy in massive hemorrhage and hemodilution. Anesthesiology. 2010;113: 1205–19. 10.1097/ALN.0b013e3181f22b5a 20881594

[39] Ogawa S, Szlam F, Bolliger D, Nishimura T, Chen EP, Tanaka KA. The impact of hematocrit on fibrin clot formation assessed by rotational thromboelastometry. Anesth Analg. 2012:115: 16–21. 10.1213/ANE.0b013e31824d523b 22467887

[40] Ogawa S, Szlam F, Chen EP, Nishimura T, Kim H, Roback JD, et al A comparative evaluation of rotation thromboelastometry and standard coagulation tests in hemodilution‐induced coagulation changes after cardiac surgery. Transfusion. 2012;52: 14–22 10.1111/j.1537-2995.2011.03241.x 21756263

[41] Solomon C, Cadamuro J, Ziegler B, Schöchl H, Varvenne M, Sørensen B, et al A comparison of fibrinogen measurement methods with fibrin clot elasticity assessed by thromboelastometry, before and after administration of fibrinogen concentrate in cardiac surgery patients. Transfusion. 2011;51: 1695–1706. 10.1111/j.1537-2995.2011.03066.x 21352237

[42] Cannesson M, Le Manach Y, Hofer CK, Goarin JP, Lehot J-J, Vallet B, et al Assessing the diagnostic accuracy of pulse pressure variations for the prediction of fluid responsiveness: a “gray zone” approach. Anesthesiology. 2011;115: 231–41. 10.1097/ALN.0b013e318225b80a 21705869

[43] Fassl J, Lurati Buse G, Filipovic M, Reuthebuch O, Hampl K, Seeberger MD, et al. Perioperative administration of fibrinogen does not increase adverse cardiac and thromboembolic events after cardiac surgery. Br J Anaesth. 10.1093/bja/aeu364 25324348

[44] Rahe-Meyer N, Hanke A, Schmidt DS, Hagl C, Pichlmaier M. Fibrinogen concentrate reduces intraoperative bleeding when used as first-line hemostatic therapy during major aortic replacement surgery: results from a randomized, placebo-controlled trial. J Thorac Cardiovasc Surg. 2013;145: S178–85. 10.1016/j.jtcvs.2012.12.083 23410777

[45] Wikkelsø A, Lunde J, Johansen M, Stensballe J, Wetterslev J, Møller AM, et al Fibrinogen concentrate in bleeding patients. Cochrane Database Syst Rev. 2013;8: CD008864 10.1002/14651858.CD008864.pub2 23986527 PMC6517136

[46] Solomon C, Hagl C, Rahe-Meyer N. Time course of haemostatic effects of fibrinogen concentrate administration in aortic surgery. Br J Anaesth. 2013;110: 947–56. 10.1093/bja/aes576 23388508 PMC3657602

[47] Momeni M, Carlier C, Baele P, Watremez C, Van Dyck M, Matta A, et al Fibrinogen concentration significantly decreases after on-pump versus off-pump coronary artery bypass surgery: a systematic point-of-care ROTEM analysis. J Cardiothorac Vasc Anesth. 2013;27: 5–11. 10.1053/j.jvca.2012.07.008 22995455

[48] Stanzel R, Henderson M, O'Blenes S. Prophylactic fibrinogen administration during complex congenital cardiac surgery leading to thrombosis of a patient's brachial artery and the cardiopulmonary bypass circuit: a case report. Perfusion. 10.1177/0267659113513312 24259497

[49] Rugeri L, Levrat A, David JS, Delecroix E, Floccard B, Gros A, et al Diagnosis of early coagulation abnormalities in trauma patients by rotation thrombelastography. J Thromb Haemost. 2007;5: 289–95. 10.1111/j.1538-7836.2007.02319.x 17109736

[50] Fries D, Innerhofer P, Perger P, Gütl M, Heil S, Hofmann N, et al Coagulation management in trauma-related massive bleeding.—Recommendations of the Task Force for Coagulation (AGPG) of the Austrian Society of Anesthesiology, Resuscitation and Intensive Care Medicine (OGARI). Anasthesiol Intensivmed Notfallmed Schmerzther. 2010;45: 552–61. 10.1055/s-0030-1265746 20839143

[51] Schöchl H, Nienaber U, Hofer G, Voelckel W, Jambor C, Scharbert G, et al Goal-directed coagulation management of major trauma patients using thromboelastometry (ROTEM)-guided administration of fibrinogen concentrate and prothrombin complex concentrate. Crit Care. 2010;14: R55 10.1186/cc8948 20374650 PMC2887173

[52] Urwyler N, Theiler L, Hirschberg M, Kleine-Brueggeney M, Colucci G, Greif R. Standard vs. point-of-care measurement of fibrinogen: potential impact on clinical decisions. Minerva Anestesiol. 2012;78: 550–55. 22310191

[53] Olde Engberink RHG, Kuiper GJAJM, Wetzels RJH, Nelemans PJ, Lance MD, Beckers EA, et al Rapid and correct prediction of thrombocytopenia and hypofibrinogenemia with rotational thromboelastometry in cardiac surgery. J Cardiothorac Vasc Anesth.2014;28: 210–16. 10.1053/j.jvca.2013.12.004 24630470

[54] Solomon C, Baryshnikova E, Tripodi A, Schlimp CJ, Schöchl H, Cadamuro J, et al Fibrinogen measurement in cardiac surgery with cardiopulmonary bypass: Analysis of repeatability and agreement of Clauss method within and between six different laboratories. Thromb Haemost. 2014;112 10.1160/TH13-12-0997 24633448

[55] Gertler R, Wiesner G, Tassani-Prell P, Braun S-L, Martin K. Are the point-of-care diagnostics MULTIPLATE and ROTEM valid in the setting of high concentrations of heparin and its reversal with protamine? J Cardiothorac Vasc Anesth. 2011;25: 981–86. 10.1053/j.jvca.2010.11.020 21315618

[56] Adam S, Karger R, Kretschmer V. Influence of different hydroxyethyl starch (HES) formulations on fibrinogen measurement in HES-diluted plasma. Clin Appl Thromb Hemost. 2010;16: 454–60. 10.1177/1076029609336855 19617247

[57] Adam S, Karger R, Kretschmer V. Photo-optical methods can lead to clinically relevant overestimation of fibrinogen concentration in plasma diluted with hydroxyethyl starch. Clin Appl Thromb Hemost. 2010;16: 461–71. 10.1177/1076029609342090 19833622

[58] Hiippala ST. Dextran and hydroxyethyl starch interfere with fibrinogen assays. Blood Coagul Fibrinolysis. 1995;6: 743–46. 8825225

[59] Skhirtladze K, Base EM, Lassnigg A, Kaider A, Linke S, Dworschak M, et al Comparison of the effects of albumin 5%, hydroxyethyl starch 130/0.4 6%, and Ringer's lactate on blood loss and coagulation after cardiac surgery. Br J Anaesth. 2014;112: 255–64. 10.1093/bja/aet348 24169821

[60] Van der Linden P, De Ville A, Hofer A, Heschl M, Gombotz H. Six percent hydroxyethyl starch 130/0.4 (Voluven) versus 5% human serum albumin for volume replacement therapy during elective open-heart surgery in pediatric patients. Anesthesiology. 2013;119: 1296–1309. 10.1097/ALN.0b013e3182a6b387 23934169

